# Silent Vanishing Lung Syndrome: Severe Emphysema in an Asymptomatic Patient

**DOI:** 10.7759/cureus.68140

**Published:** 2024-08-29

**Authors:** Cynthia Cespedes Aguirre, Leonel Gonzalez Diaz, Robert Hernandez, Afredo Gutierrez

**Affiliations:** 1 Department of Internal Medicine, Larkin Community Hospital, South Miami, USA; 2 Department of Psychiatry, Larkin Community Hospital, South Miami, USA; 3 Department of Infectious Diseases, Larkin Community Hospital, South Miami, USA

**Keywords:** paraseptal emphysema, smoking history, polysubstance use disorder, hiv infection, pulmonary bulla, silent vanishing lung syndrome

## Abstract

Vanishing lung syndrome (VLS) is an uncommon condition characterized by idiopathic giant bullous emphysema, resulting in the lungs appearing abnormally small on radiological scans. Some case reports have suggested a potential association between the development of this condition in young males, individuals with a history of heavy tobacco smoking, methamphetamine, and marijuana use, and those previously diagnosed with HIV. The primary diagnostic tools for vanishing lung syndrome include initial x-rays and high-resolution CT scans, which play a crucial role in confirming the diagnosis. The management of vanishing lung syndrome varies based on several factors, including the patient's functional status and the size and location of the bullae, with treatment options ranging from conservative approaches to surgical interventions.

In this case report, we present the case of a 42-year-old male who was a heavy tobacco smoker, had a history of methamphetamine and marijuana use, and was previously diagnosed with HIV. He initially presented to the emergency department seeking poly-substance detoxification but was incidentally found to have giant bullous emphysema on chest imaging. After stabilizing the patient, he was discharged with instructions to follow up with a pulmonologist in two months.

## Introduction

Vanishing lung syndrome, an idiopathic giant bullous emphysema, represents a rare and irreversible form of pulmonary parenchymal damage. Typically, patients afflicted by this condition exhibit a prolonged history of smoking or COPD, but occurrences have also been reported in young individuals with a background of marijuana use, HIV infection [1], or alpha-1 antitrypsin (AAT) deficiency [2]. Back in 1937, Burke documented a case of “vanishing lungs” in a 35-year-old man who experienced progressive dyspnea, respiratory failure, and radiographic and pathological evidence of giant bullae occupying a substantial portion of both hemithoraxes [3]. In this report, we highlight the case of a 42-year-old male diagnosed with HIV, who has a substantial history of marijuana use. He presented to the emergency department due to intoxication from multiple substances. During the evaluation, incidental imaging studies revealed the presence of an asymptomatic giant bullae. This discovery underscores the significance of vigilance in monitoring such unexpected findings. We will also explore treatment options [4] and potential outcomes associated with this condition.

## Case presentation

A 42-year-old male, heavy smoker, with a medical history that includes HIV, anxiety, and polysubstance use disorder, was admitted to the hospital for a detoxification process involving multiple substances. He also reported feeling fatigued, experiencing cold sensations, and sporadic coughing over the past few weeks. The patient has a recurring history of admissions to rehabilitation facilities for detoxification over the last decade and is actively managing his HIV infection with Biktarvy.

Upon admission, the patient's drug screening yielded positive results for amphetamine, cocaine, and marijuana. He also complained of fatigue, weakness, malaise, and night sweats but denied any chest pain or shortness of breath. At the time of presentation, his vital signs fell within the normal ranges. Physical examinations did not reveal any notable abnormalities, and his blood workup displayed a CD4 count of 291/mm^3^ with a CD4/CD8 ratio of 0.35. AAT level displayed a normal level of 121 mg/dL.

A chest x-ray identified a focal area of increased lucency in the right middle to lower lung, indicative of bulla formation. A chest CT scan without contrast further revealed mediastinal lymphadenopathy, which could potentially be attributed to a reactive or malignant process. Additionally, the CT scan suggested a diagnosis of vanishing lung syndrome, characterized by severe paraseptal emphysema and a large right bulla occupying over one-third of the hemithorax. Furthermore, a small pulmonary solid nodule measuring 6mm was observed in the left upper lobe. It has been advised that the patient undergoes a follow-up CT scan in six months to closely monitor these identified findings.

**Figure 1:**
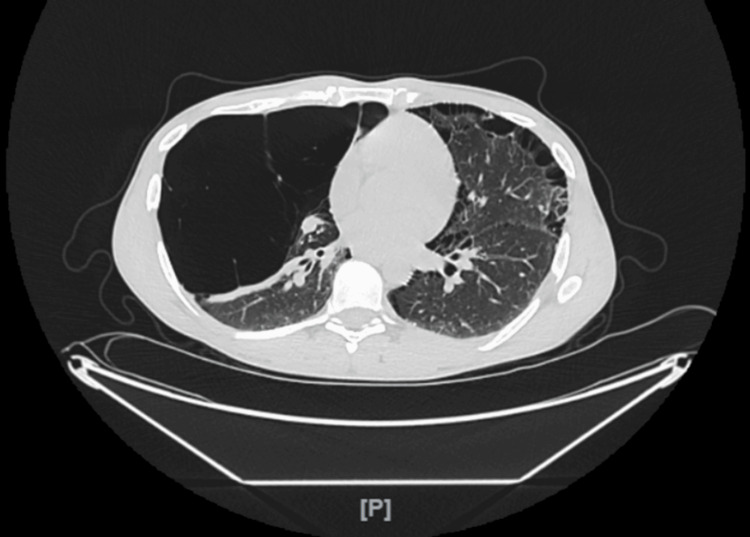
Chest CT scan transverse view showing severe bilateral apical bullous emphysema without pneumothorax.

## Discussion

Vanishing lung syndrome is a noteworthy and rare condition primarily observed in young males, often associated with factors such as smoking, marijuana abuse, HIV infection, or AAT deficiency. However, it has also been documented in non-smokers [2]. When a chest x-ray reveals the peculiar appearance of disappearing lungs or the presence of exceptionally large emphysematous bullae in such patients, vanishing lung syndrome should be considered a potential diagnosis. To confirm this syndrome, a CT chest scan is the preferred diagnostic tool, and it also serves the purpose of ruling out other pulmonary conditions, including pneumothorax [5]. The pathophysiology of Vanishing Lung Syndrome is characterized by the development of giant bullae that compress normal lung tissue, causing mediastinal shift and reduced exercise tolerance. Because Vanishing Lung Syndrome can closely mimic pneumothorax, clinicians often misdiagnose one for the other. A chest radiograph can help distinguish between the two, but high-resolution computer tomography (HRCT) of the thorax is also required for confirmation. In general, a bulla has a more rounded appearance and does not have a lung edge, while a pneumothorax has a convex pleural line.

In cases where asymptomatic patients exhibit giant bullae, conservative management like smoking cessation, nebulized bronchodilators, inhaled corticosteroids, and influenza and pneumococcal vaccination may be the appropriate course of action. However, for symptomatic patients, bullectomy stands as the definitive treatment to facilitate the re-expansion of the remaining lung tissue [6]. The postoperative prognosis is predominantly influenced by the size of the bullae and the condition of the remaining lung segments. Patients without underlying diffuse emphysema tend to experience a favorable long-term outcome. Early diagnosis and ongoing monitoring of this condition play a crucial role in preventing potentially fatal consequences. It is essential to educate patients about their condition, potential complications, and the advantages of smoking cessation and regular medical follow-up to mitigate life-threatening outcomes. Other surgical treatment options include endocavitary drainage, volume reduction with video-assisted thoracoscopic surgery, one-way endobronchial valves, or lung transplant.

## Conclusions

Vanishing lung syndrome is a condition that, while rare, is frequently observed in individuals with specific risk factors, including heavy smoking, HIV infection, marijuana usage, and young males. A comprehensive approach to diagnosis, which includes thorough history-taking, detailed physical examinations, and expertise in interpreting chest imaging, is crucial in identifying this syndrome. In cases where patients are asymptomatic and remain clinically stable, conservative management is the most suitable course of action. It involves regular follow-up with CT thorax scans, promoting smoking cessation, and providing patient education to prevent potentially life-threatening consequences. However, for symptomatic patients afflicted with giant bullae, the primary treatment approach is bullectomy, which serves as the cornerstone of therapy.
